# Determinants of Crop Diversification in Boro Rice‐Based, Wetland Farming Systems in Bangladesh

**DOI:** 10.1002/pei3.70227

**Published:** 2026-10-28

**Authors:** Mohammad Shamsul Hoq, Esrat Jahan, Moniruzzaman, Morsalina Khatun, Md. Ripon Hasan, Faria Rahman, Nazmun Nahar, Md. Imrul Kaysar, Md. Nymul Islam, Abu Hayat Md. Saiful Islam, Mohammad Ismail Hossain

**Affiliations:** ^1^ Agricultural Economics Division Bangladesh Agricultural Research Institute Joydebpur Gazipur Bangladesh; ^2^ Department of Agribusiness and Marketing Bangladesh Agricultural University Mymensingh Bangladesh; ^3^ Pulses Research Centre and Regional Agricultural Research Station BARI Ishwardi Pabna Bangladesh; ^4^ Oilseed Research Centre Bangladesh Agricultural Research Institute Joydebpur Gazipur Bangladesh; ^5^ Department of Agricultural Economics Bangladesh Agricultural University Mymensingh Bangladesh

**Keywords:** Bangladesh, crop diversification, determinants, haor region, Left‐censored Tobit and Ordered Probit model, Simpson diversification index

## Abstract

The haor landscape of Bangladesh is highly reliant on Boro rice cultivation, making farm households vulnerable to recurring floods and climate‐related risks. Crop diversification can intensify agricultural resilience and reduce production risks. This study examined the pattern, extent, and determinants of crop diversification using a cross‐sectional field survey performed in 2023 among 240 farm households in three purposively selected upazilas (Itna, Mithamoin, and Nikli) of Kishoreganj district. The Simpson Diversification Index (SDI), based on crop‐area shares, was used to measure crop diversification, while Censored Tobit and Ordered Probit models were employed to identify its determinants. The results showed that farmers were gradually familiarizing themselves with crops such as maize, mustard, jute, chili, sweet potato, groundnut, potato, pumpkin, black gram, and various vegetables, along with Boro rice. The mean SDI was 0.22, with 58.33% of households falling within the poor diversification category (0–0.24). The Tobit model indicated that household head education, farm size, agricultural training, marketing training, and extension contact positively associated with crop diversification. In contrast, road distance, duration of waterlogging, and agro‐ecological location had negative effects. The Ordered Probit results generally confirmed these associations and also revealed a positive relationship between the dependency ratio and the higher category of crop diversification. Marginal effects indicated that agricultural training, marketing training, and extension contact increased the probability of achieving excellent diversification by 6.2, 5.5, and 4.8 percentage points, respectively, while each additional kilometer from a nearby road and each additional month of waterlogging reduced this probability by 5.4 and 3.1 percentage points, respectively. These findings emphasize the importance of improving farmer training, extension services, rural infrastructure, and location‐specific waterlogging control to increase crop diversification and resilience in haor farming systems. Given the purposive selection of three upazilas in Kishoreganj district, the findings should be interpreted with caution in other haor regions.

## Graphical abstract

Crop diversification in haor areas of Bangladesh remains low and is associated with household, institutional, infrastructural, and agro‐ecological factors. Strengthening farmer socioeconomic factors, institutional access, infrastructure, and adaptation to protracted waterlogging may help greater crop diversification.

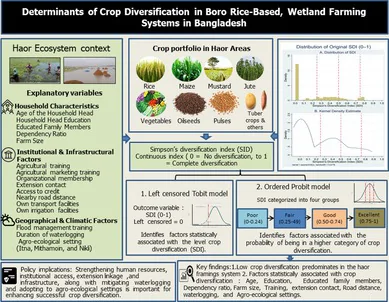

## 1 Introduction

Climate change has emerged as a key challenge for global agriculture, offering significant threats to food security, rural livelihoods, and environmental sustainability. Extreme weather events such as recurrent flash floods, rising temperatures, prolonged droughts, seasonal floods, and river erosion are impacting crop production globally, particularly in fragile and ecologically vulnerable ecosystems (IPCC 2023). Climate‐induced risks and uncertainties in crop production are very common in these regions, resulting in crop failure, unstable household income, and exposing them to greater market and production risks, particularly in developing countries where agriculture is highly climate‐sensitive (IPCC 2023; World Bank 2024). Smallholder farmers are particularly vulnerable to these risks due to their limited adaptive capacity, inadequate access to agricultural resources, and strong dependence on climate‐sensitive farming, highlighting the need for resilient farming strategies such as crop diversification (Joshi et al. 2003; IPCC 2023; Nandi et al. 2024; Akter and Mazumder 2025). Kabir et al. (2017) noted that farmers recognize the benefits of crop diversification in reducing the risk of crop failure arising from environmental uncertainties. Crop diversification has been widely used as a key risk management strategy that enhances cropping intensity, improves land and labor productivity, creates income and employment opportunities, and strengthens food and nutrition security (Thierfelder et al. 2013; Nkegbe et al. 2017; Bowles et al. 2020; Bernzen et al. 2023). In South Asia, it is increasingly important as an attempt to mitigate the adverse effects of climate change by minimizing the dependence on staple crops and optimizing the use of limited natural resources (Mandal and Bezbaruah 2013). For instance, diversification into short‐duration crops has reduced the potential risk of seasonal floods in India's flood‐prone Assam plains, while increasing food security and long‐term sustainability in Sub‐Saharan Africa and Europe (Kurdy's‐Kujawska et al. 2021). Bangladesh is one of the most vulnerable countries in the world, facing these challenges in agriculture. Policy emphasis has increased in recent decades to support crop diversification for attaining food and nutrition security and building resilience against climate‐induced vulnerabilities (Emran et al. 2022; Rahman et al. 2024; Rahman et al. 2026). It was evident from past studies that introducing new crops such as mustard, potatoes, pulses, chilies, and vegetables leads to successful diversification in challenging ecosystems like drought‐prone areas, floodplains, or coastal regions (Shopan et al. 2012; Bernzen et al. 2023). The haor ecosystem is a low‐lying wetland, with bowl‐shaped depressions consisting of around 2.00 million hectares of land and providing habitat for nearly 19.40 million people in northeastern Bangladesh. These settings are agro‐ecologically fragile, with recurring floods, flash floods, and low winter temperatures confining crop productivity and year‐round cultivation (Hoq, Raha, and Hossain 2021; Islam et al. 2023; Bokhtiar et al. 2024). The Boro‐Fallow‐Fallow is the foremost cropping pattern, with Boro rice contributing about 18% of the country's total rice production (BBS 2018). Reliance on this single crop like Boro rice exposes farmers to severe hazards, such as early flash floods frequently damage standing boro crops, groundwater‐intensive rice farming raising sustainability issues, and extended monsoon flooding leaving wide areas underutilized (Alam et al. 2021; Islam et al. 2023). Despite these limitations, the haor basin provides immense potential for crop diversification. The enormous land area in the haor remains fallow during the rabi season, pointing to untapped prospects for introducing short‐duration and high‐value crops. Evidence shows that during the rabi season, short‐duration crops such as potato, groundnut, mustard, pulses, maize, and a wide range of vegetables could be integrated into rice‐based farming systems without displacing rice cultivation (Shopan et al. 2012; Emran et al. 2022; Akter et al. 2024). Crop diversification in haor areas thus represents not only a climate adaptation strategy but also a pathway to improving income, dietary diversity, and resilience against natural calamities (Mandal and Bezbaruah 2013; Lázár et al. 2015; Bowles et al. 2020). In the literature, there have been some past studies of developed and developing countries that highlight the positive effects of crop diversification. According to Kurdy's‐Kujawska et al. (2021), small farms in Poland's frost‐hardiness zone have a positive influence on crop diversification. A study by Mandal and Bezbaruah (2013) found that crop diversity enhances farmers' income by serving as an adaptive strategy to mitigate flood risks in the Assam plains of India. Bowles et al. (2020) highlighted that long‐term crop diversification strengthens food security, promotes agricultural sustainability, and increases resilience to adverse conditions in North America. However, actual crop diversification in the haor areas is increasing with institutional support, despite the persistence of fragile ecosystems, recurrent waterlogging, and weak market linkages (Bokhtiar et al. 2024; Bernzen et al. 2023; Tisdell et al. 2019). Crop diversification is reinforced by institutional initiatives from Department of Agricultural Extension (DAE), Bangladesh Agricultural Research Institute (BARI), and Bangladesh Rice Research Institute (BRRI) through extension services, location‐specific innovations, and short‐duration crop varieties appropriate to the haor ecosystem in Bangladesh (Karim et al. 2010; Hossain et al. 2015; Kabir and Alauddin 2016; BRRI 2020). It is also prioritized in national policy outlines such as the National Agricultural Policy 2018 and the Bangladesh Delta Plan 2100 as a key strategy for climate adaptation and food security (NAP 2018; BDP 2018). Numerous studies have identified factors that influence the factors of crop diversification, including social, institutional, environmental, and geographical aspects in the worldwide (Bernzen et al. 2023). Ashfaq et al. (2008) found that land size, age, education, farming experience, off‐farm income, farm‐to‐road distance, farm‐to‐market distance, and farm machinery ownership influenced the level of crop diversification in the Punjab province of Pakistan. Nuru and Seebens (2008) also revealed that crop diversification has been significantly influenced by proximity to a town, road access, education, liquid wealth, and irrigation access in northern Ethiopia. Dessie et al. (2019) found that farmland, sex, age, and other socioeconomic factors have significantly influenced the crop diversification adopted by farmers in northwest Ethiopia. Although recent studies have significantly improved our understanding of crop diversification in Bangladesh and other developing countries, numerous important research gaps remain. Most empirical studies have focused on northern, coastal, and other conventional agricultural regions (Azad 2021; Akber et al. 2022; Nahar et al. 2024; Rashid et al. 2024; Islam et al. 2024; Rahman et al. 2024; Akter and Mazumder 2025; Rahman et al. 2026), whereas empirical evidence from the flood‐prone haor ecosystem remains scarce. Because of its distinct agro‐ecological traits and extended seasonal waterlogging, studies from other agro‐ecosystems cannot be applied directly to the haor region. Therefore, context‐specific evidence is required to support a sustainable crop diversification program. In addition, national policy reviews have revealed that crop diversification initiatives in Bangladesh has mainly emphasized on socioeconomic determinants of diversification, with insufficient emphasis on social, institutional, infrastructure, and climatic factor despite their importance in promoting sustainable diversification (Rashid et al. 2024; Nandi et al. 2024). Furthermore, most empirical research has quantified agricultural diversification using continuous indices and classical regression models, whereas only a few have employed an ordered response framework to investigate the causes of various levels of crop diversification (Piedra‐Bonilla and Cunha 2020; Bilal et al. 2026). Against this background, this study intends to answer three research questions: (i) what cropping practices exist and the level of crop diversification in the haor areas of Bangladesh? (ii) Which household, institutional, infrastructural, and agro‐ecological factors are associated with crop diversification? and (iii) How do these factors effect farmers' likelihood of attaining different levels of crop diversification? Therefore, this study addresses these gaps by investigating the determinants of crop diversification among farm households in the haor areas of Bangladesh using an ordered probit model while jointly considering household characteristics, institutional support, infrastructural accessibility, and agro‐ecological factors.

## 2 Materials and Methods

### 2.1 Description of the Study Area

The haor areas of Bangladesh were located in the northeastern region of Bangladesh. The region is also ecologically vulnerable due to a flood‐prone ecosystem. Among the seven haor districts, Kishoreganj is the most potential area for diversified crop cultivation beyond rice cultivation. The district has 13 Upazillas; of these, four (Austagram, Itna, Mithamoin, and Nikli) are completely encircled by haor, while five (Bajitpur, Tarail, Karimgonj, Kuliarchar, and Bhairab) are only partially encircled. In total, 97 haors are in Kishoreganj district (Prosoil Foundation Consultant Bangladesh 2016). The Old Brhamaputra, Fuleshwari, Ghorauttra, Meghna, Dhanu, Baulai, Norshunda, Sonai, Mogra, Baro Chinai, Singua, Suti, Arial Kha, Soaijani, Kali, Kula, etc. rivers are passed through the Kishoreganj district, which is the root cause of livelihood vulnerability (Quddus 2014; Hoq, Raha, and Hossain 2021) and barriers to diversifying farming activities. The district has 13 upazilas (administrative sub‐units), and three administrative sub‐units (upazilas), that is, Itna (21 haors), Mithamoin (16 haors), and Nikli (19 haors), were purposively selected based on dense *haor* area to study the existing crop diversity. The single‐cropped area in haor districts increased from 24% to 44% between 2007 and 2021, primarily due to the transformation of previously uncultivated land into cropland. Between 2013 and 2020, approximately 88,663 ha of fallow land were brought under cultivation, contributing to this growth. This trend suggests potential crop diversification and increased cropping intensity in the haor region (Bokhtiar et al. 2024). The Bangladesh Agricultural Research Council (BARC) provided administrative and geographic reference data for the study location. Using these geographical data, Kishoreganj district and the selected upazilas were delineated and visualized with QGIS version 3.44.0 software. The geographical location of Kishoreganj District and the spatial distribution of the study areas are shown in Figure 1A,B, respectively.

**FIGURE 1:**
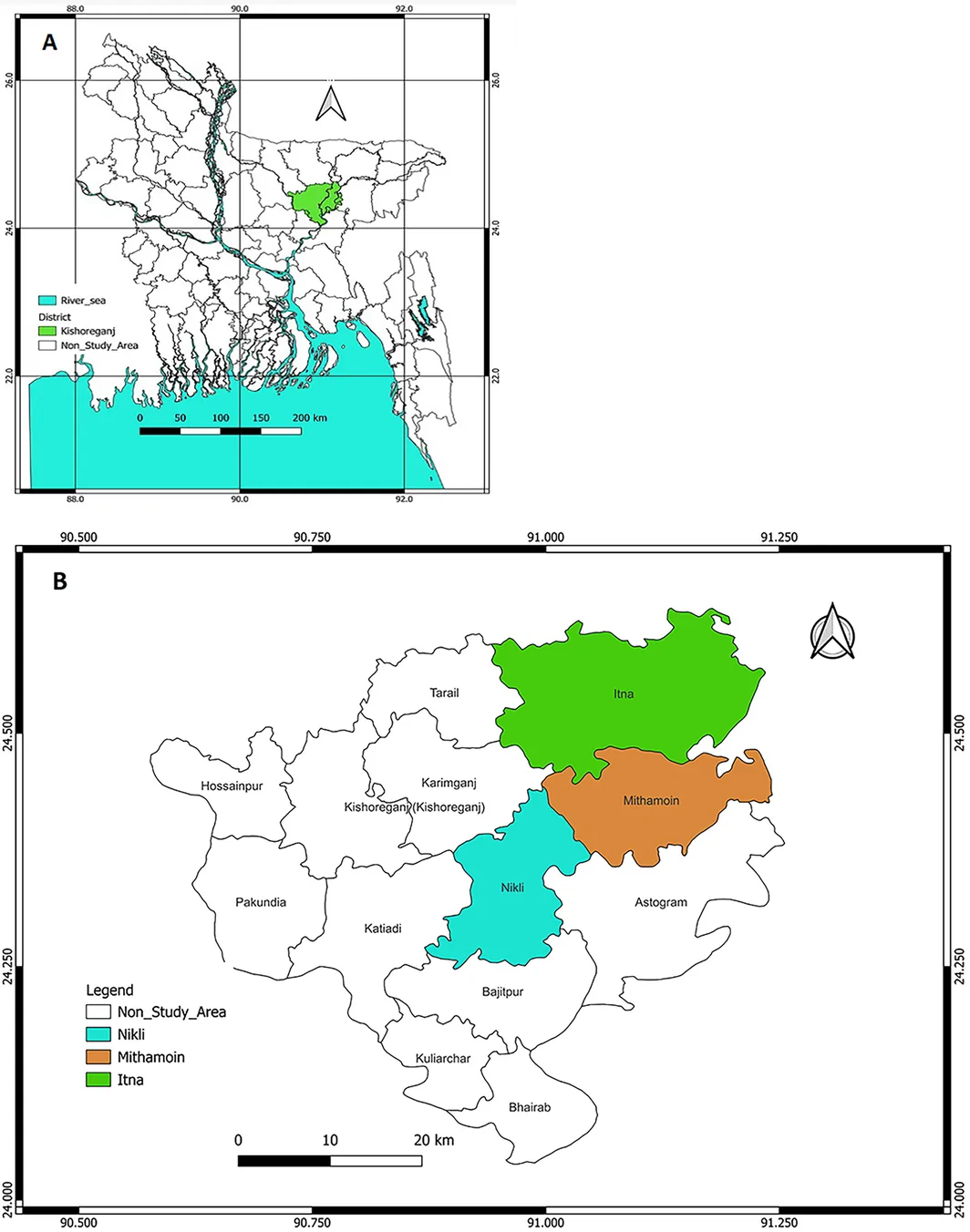
(A) Shows the Kishoreganj district in Bangladesh map, and (B) Shows the selected upazilas in Kishoreganj district.

### 2.2 Sample Size and Sampling Procedure

A multistage sampling technique was used to select the study areas and respondents. In the first stage, Kishoreganj district was purposively chosen because it represents one of the key haor regions of Bangladesh, where agriculture is mostly focused on Boro rice farming and has significance for crop diversification. In the second stage, three upazilas, Itna, Mithamoin, and Nikli, were purposively selected for their agro‐ecological traits, prolonged waterlogging, and relevance in haor agriculture. Thirdly, one or two villages were randomly picked from each upazila to ensure that every village had an equal chance of being included in the study. Finally, a list of farmers was obtained with the help of the Department of Agricultural Extension (DAE), and 240 sample crop farmers were randomly picked from the sampling frame by using the Equation (1) given by Kothari (2004):

(1) $$n=\frac{p\times \left(1-p\right)\times z^{2}}{e^{2}}$$

$$n=\frac{0.5\left(1-0.5\right)\times 1.96^{2}}{0.065^{2}}=\frac{0.9604}{0.004225}=227\approx 240$$

where *n* denote the total sample size and *p* is the proportion of respondents whose value was 0.5 to get a maximum number of the respondents. *z* stands for the standard error in the 95% confidence interval, which is 1.96, and *e* is the margin of error that the researchers are willing to accept, which is found to be 6% by Hoq et al. (2022) and 5.6% by Sujan et al. (2026). Thus, the 240 sample households were picked proportionally from study areas (Table 1). Because of this purposive geographic selection, the findings of this study are limited to the selected upazilas and should not be applied to the entire haor basin without caution.

**TABLE 1:** Determination of sample size.

District	Upazila	Total households (*N*)	Sample households (*n*)	Percent
Kishoreganj	Nikli	20,762	70	29
	Itna	33,310	110	46
	Mithamoin	17,600	60	25
	Total	71,672	240	100

*Source:* DAE (2023).

### 2.3 Data Sources

#### 2.3.1 Primary Data Collection

Primary data were collected through a field survey conducted between February and April 2023 using a structured questionnaire developed after a rigorous review of the literature relevant to the objectives of the study. The questionnaire was pre‐tested before being used for face‐to‐face interviews with the household head in their native language (Bangla) who is primarily responsible for crop production decisions. The data were collected by three trained enumerators working under the supervision of the principal investigator to ensure consistency and data reliability. The study's objectives were explained to respondents before each interview, and verbal informed consent was obtained. All 240 selected households took part, resulting in a 100% response rate. The completed questionnaires were checked for completeness and consistency before data entry, and no missing observations were found in the final dataset. The supporting dataset and analytical materials are provided as Supporting Information (Files S1 and S2). Both files are also openly available through the Zenodo repository (Hoq et al. 2026, Version 3; https://doi.org/10.5281/zenodo.23375916). Statistical analyses were performed using Excel and Stata version 15 software.

#### 2.3.2 Secondary Data Sources

Secondary data were collected from a variety of credible national and international sources to supplement primary data and provide contextual information for the study. These secondary data were obtained from various reports, journal articles, books, documents, printed materials, official documents, statistical publications, and other relevant online resources. Secondary data were reviewed, verified, and synthesized to reinforce primary data analysis, validate research findings, and provide a thorough understanding of crop diversification systems and climate risks.

### 2.4 Conceptual Framework

The conceptual framework shown in Figure 2 was developed to determine the level, extent, and determinants of Boro rice‐based crop diversification in the wetland ecosystem of Bangladesh. Crop diversification is associated with the interplay of household, institutional, infrastructural, and agro‐ecological factors that collectively shape farmers' production decisions. During the dry Rabi season, approximately 80% of the cultivated land in the haor basin of Bangladesh is devoted to a single Boro rice crop, while only about 10% is used for T. Aman rice cultivation (Alam et al. 2010; Hoq, Udddin, et al. 2021). This framework provides emphasis on crop diversification to reduce vulnerability of haor households, because crop diversity is basically recognized as a risk reduction strategy and income diversification from various crops (Dessie et al. 2019). Simpson's Diversification Index (SDI) was used to assess crop diversification and was used as the dependent variable in the Censored Tobit model to identify crop diversification‐related factors (Dessie et al. 2019). The sample farmers' SDI is further classified into four ordinal measures: poor (0–0.24), fair (0.25–0.49), good (0.50–0.74), and excellent (0.75–1.00) level of diversification, and it is used as a dependent variable in an ordered probit model to determine the factors that influence the level, extent, and magnitude of crop diversification (Nkegbe et al. 2017; Ahmed et al. 2018; Piedra‐Bonilla and Cunha 2020).

**FIGURE 2:**
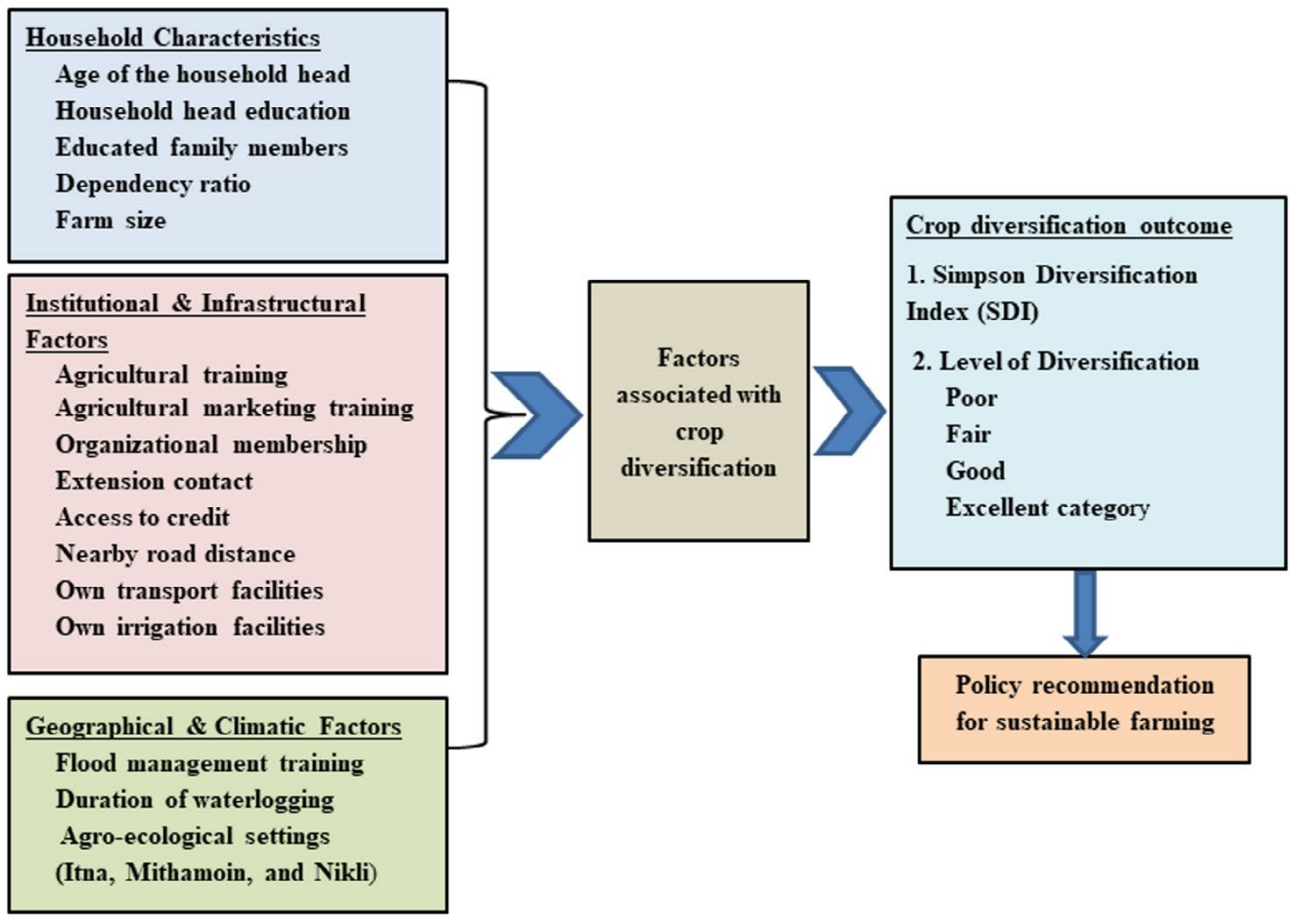
Conceptual framework illustrating the determinants of crop diversification in the haor ecosystem of Bangladesh.

### 2.5 Model Specification

#### 2.5.1 Simpson's Diversification Index

Different methods were used for estimating household crop diversification, such as the Simpsons Diversification Index (SDI), Herfindahl, Gibbs, and Martin's indexes, etc. (Alemu 2023) but Simpson's Diversification Index (SDI) is best to show the share of diversified crops in gross cropped area (Agyeman et al. 2014). The household crop diversification index was calculated by using the following equation as done by Chilongo (2014):

(2) $${\text{Simpson}}^{\prime }s\;\text{Index of Diversity}\;\left(\mathrm{SID}\right)=1-\sum_{i}^{n}P_{i}^{2}$$

where *P*_*i*_ = proportion of the *i*th crop in the gross cropped area.

(3) $$\;P_{i}=\frac{A_{i}}{\sum_{i}^{n}A_{i}}$$

where *A*_*i*_ = area under the *i*th crop.

$$\sum_{i}^{n}A_{i}=\text{Gross crop Area},i=1,2,3,\ldots ,n\;\left(\text{number of crops}\right).$$

The index ranges from 0 to 1, where 0 refers to no diversification and 1 refers to the highest level of diversification.

#### 2.5.2 Left‐Censored Tobit Regression Model

To analyze the determinants of crop diversification of haor farm households, a Left‐censored Tobit regression model was employed. The dependent variable, Simpson Diversification Index (SDI), ranges from 0 to 1, with a substantial proportion of observations taking the value zero. Since the dependent variable is censored at the lower limit, the Ordinary Least Squares (OLS) estimator is inappropriate because it ignores the censoring of the dependent variable. Therefore, the Tobit model was applied to estimate the factors associated with both the decision to diversify crops and the level of crop diversification (Greene 2003; Wooldridge 2016; Alemu 2023).

The latent Tobit model is specified as:

(4) $${{\mathrm{SDI}}^{*}=y}_{i}^{*}=x_{i}\beta +\varepsilon_{i}$$

where $y_{i}^{*}$ = Latent (unobserved) of Simpson Diversification Index (SDI*) or the variable of primary interest, $\;x_{i}$ = Vector of explanatory variables for the *i*th household, β = Vector of unknown parameters to be estimated, $\varepsilon_{i}$~ N (0, *σ*2).

The observed SDI is defined as:

(5) $$\;y_{i}=\left\{\begin{array}{c} 0,\;\text{if}\;y_{i}^{*}\leq 0\\ y_{i}^{*},\;\text{if}\;y_{i}^{*}>0 \end{array}\right\}$$

The empirical Tobit model does not require the specification of an upper censoring point since no observation is censored at 1. The Tobit parameters were estimated using the Maximum Likelihood Estimation (MLE) method. Tobit coefficients do not directly represent the effects of the explanatory variables on the observed SDI, so average marginal effects were calculated for ease of interpretation. The marginal effects indicate the expected change in the observed Simpson Diversification Index (SDI) associated with changes in the explanatory variables, while holding all other factors constant and accounting for the left‐censoring of the SDI at zero. The expected value for the observed dependent variable is expressed as:

(6) $$E\left(y_{i}|x_{i}\right)=\Phi \left(\frac{x_{i}\beta }{\sigma }\right)x_{i}\beta +\sigma \phi \left(\frac{x_{i}\beta }{\sigma }\right)$$

where $\Phi \left(\cdot \right)$ = Cumulative distribution function (CDF), $\phi \left(\cdot \right)$ = probability density function (PDF) of the standard normal distribution.

The marginal effect of the *k*th explanatory variable on the expected value of the observed SDI is

(7) $$\frac{\partial E\left(y_{i}|x_{i}\right)}{\partial x_{\mathrm{ik}}}=\Phi \left(\frac{x_{i}\beta }{\sigma }\right)\beta_{k\;}$$

where *β*_*k*_ denotes the coefficient of the *k*th explanatory variable. The average marginal effects were estimated using the $\text{margins},\text{dydx}\left(*\right)\;\text{predict}\left(\text{ystar}\left(0,.\right)\right)$ command in Stata 15 software.

#### 2.5.3 Ordered Probit Model

The estimated Simpson Diversification Index of the sample farmers is further transformed into four categorical and ordinal measures. In this case, Ordered Probit or Logit models are the most appropriate techniques for analysis of such ordered discrete variables (Maddala 1983). Multinomial logit or probit models would not be appropriate for the ordinal types of the dependent variable (Greene 2003; Maddala 1983). The Probit model assumes a normal distribution of the error term, whereas the logit assumes a logistic distribution. In practice, the logistic and normal distributions produce similar results. Nonetheless, the ordered probit model is one of the most commonly used approaches for ordered dependent variables (Nkegbe et al. 2017). As a result, the Ordered Probit is the best‐fitting model in this investigation, and the model is as follows.

(8) $$y_{i}^{*}=X_{i}^{\prime }\beta +\varepsilon_{i},\;i=1,2,3,\ldots ,N$$

where $y_{i}^{*}$ is supposed to be a dependent unobservable random variable, while treating $y_{i}$ is the observed categorical variable with “*j*” response categories and also as a proxy for the unobserved latent random $\;y_{i}^{*}$. The latent variable $\;y_{i}^{*}$ is the hypothesized predicator of the crop diversity index, β is a vector of parameters to be estimated, and $\varepsilon_{i}$ is an error term which is assumed to be normally distributed.

The relationship between the values of the observed variable $y_{i}$ and the unobserved latent variable $y_{i}^{*}$ is assumed to be as follows:

(9) $$y=j,\;\text{if}\;\mu_{j}-1<y_{i}^{*}\leq \mu_{j},\;j=1,2,3,\ldots ,j$$

where *μ* indicates the unknown threshold parameters, $\mu -1=-\infty ,\;\mu_{0}=0,\mu_{j}=\infty$ and the estimated cut‐off points, μ follows the order $\;\mu -1<\mu_{0}<\mu_{1}\cdots <\mu_{j}$.

The probabilities that a given household will fall within a response category of *j* follow:

(10) $$\begin{array}{c} P_{\mathrm{ij}}=P\;\left(y_{i}=j\right)=P\;\left(u_{j}-1<y_{i}^{*}\leq u_{j}=F(u_{j}-X_{i}^{\prime }\beta \right)-F\left(u_{j-1}-X_{i}^{\prime }\beta \right) \end{array}$$

where $F^{\prime }\left(.\right)$ is the standard normal cumulative distribution function, and *j* is the response categories, in this Cases 1, 2, 3, and 4, since the extent of crop diversification categorized into four response category. Crop diversification level is affected by changes in the explanatory variables, and the consequences are measured by marginal effects. As a result, the marginal effect of an increase in a regressor *x* on the likelihood of a household falling within j response category is as follows:

(11) $$\begin{array}{c} \frac{{\partial P}_{\mathrm{ij}}}{{\partial x}_{\mathrm{ri}}}=\left\{F^{\prime }\left(U_{j-1}-X_{i}^{\prime }\beta \right)-F^{\prime }\left(U_{j}-X_{i}^{\prime }\beta \right)\right\}=\left\{F^{\prime }\left(U_{j-1}-X_{i}^{\prime }\right)-F^{\prime }\left(U_{j}-X_{i}^{\prime }\right)\right\}\beta \end{array}$$

where *F*′(·) is the standard normal density function. Then, following (Nkegbe et al. 2017), the final estimated model is specified as:

(12) $${\text{CD}}_{\text{ij}}=\alpha +\beta X_{i}+\varepsilon_{i}$$

where CD is crop diversification; subscript *i* (*i* = 1, 2, 3, ⋯, *N*) denotes the number of households, subscript *j* (*j* = 1, 2, 3, 4) denotes the crop diversity categorization of Simpson Diversification Index; *X* represents explanatory variables used in the model; *α* and *β* are the unknown parameters to be estimated and *ε* ≈ NID (0, 1). Finally, the maximum likelihood technique was used to estimate the values for the parameters.

### 2.6 Diagnostic Tests for Model Specification

We examined multicollinearity among the independent variables using both a correlation matrix and the variance inflation factor (VIF). The findings indicate that there is no strong multicollinearity, since the mean VIF was 1.314 and the individual VIF values were between 1.065 and 1.681, all considerably below the acceptable cutoff of 10 (Wooldridge 2016). The tolerance values (1/VIF) were also higher than 0.20, which means that multicollinearity is not a problem in the estimated model (Appendix Table A1). The correlation coefficients among the explanatory variables varied from low to moderate, with none exceeding the critical threshold of 0.70. Therefore, all explanatory variables were retained for subsequent analysis (Gujarati 2009; O'Brien 2007) (Appendix Table A2). Potential heteroskedasticity was assessed using Breusch‐Pagan/Cook‐Weisberg and White tests based on an auxiliary OLS specification using the same outcome and explanatory variables. The two tests provided conflicting evidence for the constant‐variance assumption. To obtain more reliable statistical inference under potential heteroskedasticity, we used heteroskedasticity‐robust standard errors in both the Tobit and Ordered Probit estimations (Appendix Table A3).

### 2.7 Explanatory Variables Used in the Model

The explanatory variables were classified into three categories: household characteristics, institutional and infrastructural factors, and geographical and climatic factors (Hoq et al. 2022; Khan et al. 2017). Table 2 displays a list of explanatory variables and their definitions. These variables were used as covariates in the Censored Tobit and Ordered Probit models to investigate the factors that influence the degree and magnitude of crop diversification in the haor region of Bangladesh.

**TABLE 2:** Definition of variables, their measurement unit and sources.

Variables name	Definition	Sources
Household characteristics		
Age of the HH	Household head's age in years	Dessie et al. (2019)
Education of the HH	Household head's years of schooling	Islam et al. (2017) and Dessie et al. (2019)
Educated family member	The number of educated member in the household	Hoq et al. (2022)
Dependency ratio	It is a ratio of the population < 15 and > 65 years of age to the working‐population between 15 and 65 years of age	Hoq et al. (2022)
Farm size	The total agricultural land of per household in hectare	Dessie et al. (2019) and Alemu (2023)
Institutional and infrastructural factors		
Agricultural training	Whether the household head or another household member received agricultural training (Yes = 1, No = 0)	Hoq et al. (2022)
Agricultural marketing training	Whether the household head or another household member received training in agricultural product marketing (Yes = 1, No = 0)	Dube and Guveya (2016)
Organizational membership	Whether the household head or any household member is a member of an organization (Yes = 1, No = 0)	Islam et al. (2017)
Extension contact	Whether the respondent received general advice from an agricultural extension officer (Yes = 1, No = 0)	Appiah‐Twumasi and Asale (2024) and Dessie et al. (2019)
Access to credit	Whether the respondent has access to institutional credit facilities (Yes = 1, No = 0)	Islam et al. (2017) and Dessie et al. (2019)
Nearby road distance	The distance from home to nearby road in KM	Islam et al. (2017)
Own transport facilities	Whether the household head has own transport facilities (Yes = 1, No = 0)	Hoq, Udddin, et al. (2021)
Own irrigation facilities	Whether the household head has own irrigation facilities (Yes = 1, No = 0)	Dube and Guveya (2016)
Geographical and climatic factors		
Flood management training	Whether the household head or any other member of the household have training on flood management (Yes = 1, No = 0)	New
Duration of waterlogging	Average number of months that the land remained waterlogged	Hoq, Udddin, et al. (2021)
Agro‐ecological settings	Household located in different agro‐ecological zone (1 = Nikli upazila, 2 = Mithamoin upazila, and 3 = Itna upazila)	Hoq, Udddin, et al. (2021)

## 3 Results and Discussions

### 3.1 Descriptive Statistics

The descriptive statistics of the sample respondents are depicted in Table 3, offering valuable insights into their household characteristics, infrastructural and institutional factors, as well as geographical and climatic conditions. The household characteristics indicate that the mean age of household heads was 46.83 years, implying that the majority of respondents are falling into the economically productive age group. Similar results have been observed in rural household studies in South Asia and Sub‐Saharan Africa, where middle‐aged farmers predominantly influence agricultural decision‐making (Diiro 2013; Tura et al. 2010). The average education level of household heads was 5.15, which is close to the national average in rural Bangladesh. This result shows that the educational level is low but becoming better compared to prior years (BBS 2022). The average number of educated family members was 2.43, highlighting the significance of household human capital in agricultural practices, which is consistent with findings from Ethiopia (Asfaw and Admassie 2004). The average dependency ratio was 0.63, indicating a moderate level of work support for each family. The average farm size was 1.52 ha, which is more than the 0.52 ha national average (BBS 2019). This is because there is typically more land available in the haor areas due to the large floodplains and lower population density. Smallholders still control the majority of farming, though, as they do in many developing nations (FAO 2014). The institutional and infrastructural factors show that around 33% of households had access to agricultural training, 15% had agricultural marketing‐related training, 19% belonged to at least one formal organization, and 65% received extension services. These values indicate a lack of institutional support, which is consistent with earlier studies in Bangladesh (Rahman and Salim 2013) and comparable evidence from Kenya and Ghana (Anderson and Feder 2007; Ragasa et al. 2013), where access to training and extension is uneven. About 33% of households reported having access to credit, which is comparable to microfinance penetration levels in rural Bangladesh (Khandker 2005). A similar pattern of credit availability has also been found among smallholder farming communities in Vietnam and Nepal (Devkota and Upadhyay 2013; Hoang et al. 2020). Households were 0.53 km away from the closest road on average, indicating moderate access to road infrastructure, which has been found to influence farmers' decisions to diversify crops (Inoni et al. 2021; Melak et al. 2021). However, just 10% reported having their own transport facilities, while 28% owned irrigation facilities, indicating constraints in mechanization. The geographical and climatic factors indicate that only 18% household obtained flood management training and they faced an average of 5.69 months of waterlogging each year, exposing the climate‐vulnerable nature of the haor region to periodic flash, exposing the climate‐vulnerable nature of the haor region to recurring flash flood. These findings are consistent with previous findings from Bangladesh's flood‐prone areas (Bahauddin and Uddin 2012; Islam et al. 2020; Hoq et al. 2022), which emphasized that extended waterlogging accelerates livelihood risks in haor areas. Agro‐ecological settings indicate that 25% of respondents originated from Mithamoin and 46% from Itna upazila; households in haor basins have prominent risks of crop failure, income fall, and displacement. Overall, the descriptive statistics show that smallholder households have limited human and physical assets, limited access to institutional services, and a lot of exposure to climate‐related risks. These scenarios are consistent with both the national context of Bangladesh and international evidence from other developing countries.

**TABLE 3:** Descriptive statistics of the explanatory variables.

Variable	Mean	SD	Min	Max
Household characteristics				
Age of the HH	46.83	12.34	17	75
Education of the HH	5.15	3.96	0	14
Educated family member	2.43	1.55	0	8
Dependency ratio	0.63	0.71	0	5
Farm size	1.52	1.33	0.19	7.37
Infrastructural and institutional factors				
Agricultural training	0.33	0.47	0	1
Agricultural marketing training	0.15	0.36	0	1
Organizational membership	0.19	0.39	0	1
Extension contact	0.65	0.48	0	1
Access to credit	0.33	0.47	0	1
Nearby road distance	0.53	0.58	0	3
Own irrigation facilities	0.28	0.45	0	1
Own transport facilities	0.10	0.29	0	1
Geographical and climatic factors				
Flood management training	0.18	0.38	0	1
Duration of waterlogging	5.69	0.72	3	7
Agro‐ecological settings				
Mithamoin	0.25	0.43	0	1
Itna	0.46	0.50	0	1

### 3.2 Household's Existing Crop Diversification Status

Figure 3 depicts the percentage of farmers who cultivate various crops in the haor areas of Bangladesh. Almost all respondent farmers (100%) produced Boro rice ( *Oryza sativa* ), indicating its dominance as the principal crop in the haor areas. In addition to Boro rice, farmers cultivated a variety of other crops, but in small quantities. These covered maize ( *Zea mays* ) (17.50%), mustard (*Brassica* spp.) (14.58%), jute (*Corchorus* spp.) (10.42%), green chili ( *Capsicum annuum* ) (9.17%), sweet potato ( *Ipomoea batatas* ) (8.75%), groundnut ( *Arachis hypogaea* L.) (7.50%), potato ( *Solanum tuberosum* L.) (7.08%), pumpkin (*Cucurbita moschata Duchesne*) (6.67%), black gram ( *Vigna mungo* ) (6.25%), cucumber ( *Cucumis sativus* ) (5.83%), bitter gourd ( *Momordica charantia* ) (5.00%), snake gourd ( *Trichosanthes cucumerina* ) (4.58%), brinjal ( *Solanum melongena* ) (4.17%), and tomato ( *Solanum lycopersicum* ) (4.17%). These results showed that crop diversification is still low in the haor areas since Boro rice cultivation predominates in farming systems. The small proportion of farmers cultivating other crops indicates a relatively restricted cropping pattern, which is perhaps influenced by prolonged seasonal flooding, a single‐cropping pattern, and agro‐ecological conditions that favor Boro rice cultivation. These findings are consistent with the study conducted by Islam et al. (2023) in Dingaputa haor of Netrakona District, Bangladesh. They found that only 10.8% of farmers are engaged in diversified crop production like oilseeds, pulses, and vegetables other than Boro rice cultivation. Although crop diversification is still low, it makes a good start towards a more diversified farming system in the haor areas (Shopan et al. 2012; Islam et al. 2023). Basically, producers adopt it as a risk reduction strategy and income diversification (Pacheco et al. 2018; Thapa et al. 2018; Bernzen et al. 2023).

**FIGURE 3:**
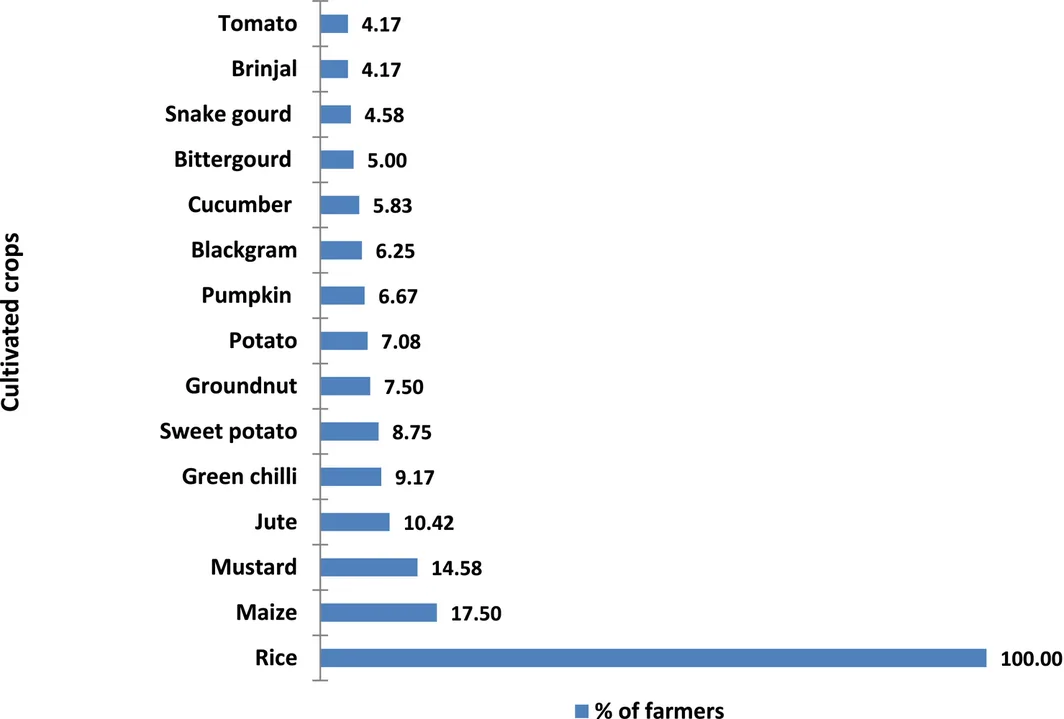
Existing crop diversification status in the haor area.

Table 4 indicates the extent and magnitude of crop diversification obtained from the Simpson Diversification Index. According to the SDI index, the mean crop diversification index is 0.22, implying that farm households are poorly diversified in the haor areas of Bangladesh. Though it is poor, it is encouraging for the Boro rice‐dominating farming system in the haor areas. This monotonous proclivity of rice cultivation is supported by Azad (2021) and Nasim et al. (2017). It is evident from Table 4 that 58.33% of the households having indices between (0–0.24) which suggest that most of the farmers are poorly diversified. Islam et al. (2023) also found similar evidence favoring poor crop diversification in haor areas. The fairly diversified category (0.25–0.49) includes about 23.76% of households, followed by 9.58% in the good category (0.50–0.74) and 8.33% in the excellent category (0.75–1). These results are consistent with the findings of Azad (2021) and Nahar et al. (2024) in Bangladesh, as well as Aheibam et al. (2022) in India, who reported similar patterns of crop diversification among farm households.

**TABLE 4:** Extent and magnitude of crop diversification index.

Extent of crop diversification	No. of farmers	% of farmers
Poor (0–0.24)	140	58.33
Fair (0.25–0.49)	57	23.76
Good (0.50–0.74)	23	9.58
Excellent (0.75–1)	20	8.33
Total (0–1)	240	100.00
Average crop diversification index	0.22	

*Source:* Field survey 2023.

Figure 4A shows the distribution of the original SDI across the sampled households, while Figure 4B presents its kernel density estimate. Both plots show a strong concentration of observations at the lower end of the SDI scale, indicating that crop diversification was generally low among the sampled households. The figures provide a visual illustration of the continuous SDI distribution and complement the descriptive statistics reported in the study. The distribution provides additional information on the continuous outcome beyond the four‐category classification used in the Ordered Probit analysis.

**FIGURE 4:**
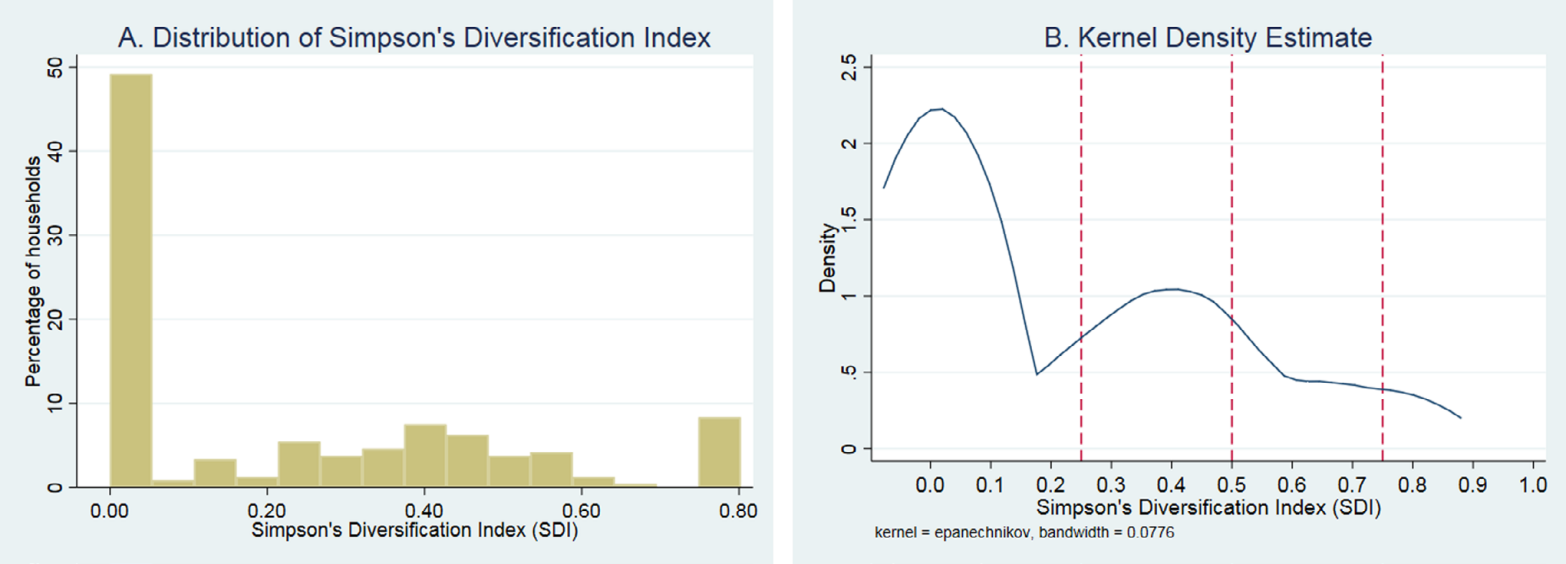
(A) Distribution of the Simpson's Diversification Index (SDI) among sampled households, and (B) Kernel density estimate of the SDI distribution.

### 3.3 Determinants of Crop Diversification: A Left‐Censored Tobit Model

The parameter estimates of the left‐censored Tobit model are summarized in Table 5. The left‐censored Tobit model was statistically significant overall (*F*(17, 223) = 11.21, *p* < 0.001), suggesting a significant relationship between the explanatory variables and the observed Simpson Diversification Index (SDI). The model was estimated using 240 observations, including 122 uncensored and 118 left‐censored observations, with no right‐censored observations. The model produced a log pseudolikelihood of −98.201 and a pseudo *R* ^2^ of 0.399. The analysis sketched some significant explanatory variables that influenced households' decision to diversify their crops. To facilitate interpretation, both the Tobit coefficients and the average marginal effects are reported. While the Tobit coefficients measure the effects of explanatory variables on the latent propensity for crop diversification, the average marginal effects represent the expected change in the observed Simpson Diversification Index (SDI) caused by a one‐unit change in each explanatory variable, while other factors remain constant. Among the household characteristics employed in the study, household head education and farm size were found to be significant with a positive sign at both stages (parameter co‐efficient and its marginal effect estimates) of the Tobit regression. The positive and statistically significant coefficient of the household head education implies that educated households are more likely to participate in non‐rice crop production. It is clear that educated household heads, those with primary, secondary, and higher secondary education, understand the benefits of modern technologies and equipment, which motivates them to grow a diverse crop on their farm. That means that one additional year of schooling for the household head increased the expected crop diversity by 1%. This finding is consistent with Nahar et al. (2024). On the other hand, significant farm size implied that households with large farms are more likely to cultivate diverse crops. The average marginal effect showed that a one‐hectare increase in farm size increased the predicted crop diversity index by 4.3%. This finding is consistent with Gebiso et al. (2023) and Makate et al. (2016), who pointed out that larger farm holdings increase crop diversity by enabling farmers to cultivate a wider range of crops, lowering production risk and increasing farm profitability. Among the institutional and infrastructural factors, agricultural training, agricultural marketing training, and extension contact had a significant positive influence on crop diversification, whereas nearby road distance had a significant negative influence. Agricultural training had a significant positive association with crop diversification, implying that trained farmers have the knowledge and technical skills required to implement improved production practices and diversify their cropping systems (Dey 2020). Similarly, agricultural marketing training intensely associated with crop diversification by boosting farmers' grasp of market demand, price information, and marketing prospects, resulting in a wider range of crops cultivation. The average marginal effects of agricultural training and agricultural marketing training were 0.089 and 0.068, respectively, which were statistically significant, indicating that participation in both training programs increased the expected crop diversification index by 8.9% and 6.8%, while other factors remained constant. These findings are similar to those of Dey (2020) and Mamun et al. (2026), who emphasized the importance of agricultural training and commercialization in encouraging crop diversification. Access to extension contact is positively associated with smallholder farmers' crop diversification decisions because it can provide a consistent supply of technical knowledge on crop management practices and other pertinent production information (Mango et al. 2015). Farmers with access to extension services are expected to adopt an 8.4% more varied cropping system than those who do not (Makate et al. 2016; Mamun et al. 2026). The study found that crop diversity is inversely related to road distance. Farmers who live farther away from the roadside produce fewer crop species. Each additional kilometer of distance between the household and the nearest road was associated with a 10.5% lower level of crop diversification (Hoq, Udddin, et al. 2021). Maru et al. (2022) also found that farmers who lived near highways were more likely to grow multiple crops and participate in marketplaces than farmers who were located farther away. Geographical and climatic factors were found to be significantly associated with crop diversification; prolonged waterlogging was negatively associated with diversification, while agro‐ecological settings were positively correlated (Khan et al. 2017). The negative association between waterlogging and crop diversification indicated that households experiencing prolonged waterlogging tended to have lower levels of crop diversification. The marginal effect indicated that an additional month of waterlogging was associated with a 4.7% lower expected level of crop diversification (Hoq et al. 2022). Households located in Mithamoin upazila had significant negative association with crop diversification than those in Nikli upazila. The average marginal effect indicated that households in Mithamoin had 7.6% less expected crop diversity than those in Nikli. This could be due to the more widespread and prolonged waterlogging in Mithamoin, which restricts the cultivation of non‐rice crops and limits their opportunities to diversify beyond the Boro rice‐based cropping system. This finding is consistent with Dube and Guveya (2016) and Hoq et al. (2022), who reported that unfavorable agro‐ecological conditions and environmental constraints reduce farmers' capacity for crop diversification.

**TABLE 5:** Left‐censored Tobit regression results of crop diversification.

Crop diversification	Co‐efficient	Robust SE	*p*	*dy*/*dx*	SE	*p*
Household characteristics						
Age of the HH	0.002	0.002	0.347	0.001	0.001	0.345
Education of the HH	0.018***	0.007	0.009	0.010***	0.004	0.008
Educated family member	−0.017	0.016	0.306	−0.009	0.009	0.305
Dependency ratio	0.043	0.030	0.151	0.024	0.017	0.149
Farm size	0.076***	0.016	0.000	0.043***	0.009	0.000
Infrastructural and institutional factors						
Agricultural training	0.160***	0.059	0.007	0.089***	0.033	0.007
Agricultural marketing training	0.122**	0.061	0.046	0.068**	0.034	0.046
Organizational membership	0.009	0.062	0.885	0.005	0.034	0.885
Extension contact	0.151**	0.060	0.012	0.084**	0.033	0.011
Access to credit	0.069	0.052	0.193	0.038	0.029	0.190
Nearby road distance	−0.189***	0.061	0.002	−0.105***	0.034	0.002
Own irrigation facilities	0.035	0.051	0.485	0.020	0.028	0.483
Own transport facilities	0.047	0.074	0.526	0.026	0.041	0.525
Geographical and climatic factors						
Flood management training	−0.036	0.060	0.549	−0.020	0.033	0.550
Duration of waterlogging	−0.084***	0.030	0.006	−0.047***	0.017	0.005
Agro‐ecological settings	Nikli upazila is a base category					
Mithamoin	−0.144**	0.067	0.032	−0.076**	0.035	0.031
Itna	−0.010	0.058	0.862	−0.006	0.034	0.862
Constant	0.203	0.227	0.372			
Model fit statistics						
Number of observation	240					
Uncensored	122					
Left‐censored	118					
Right‐censored	0					
Log pseudolikelihood	−98.201					
*F*(17, 223)	11.21					
Prob > *F*	0.00					
Pseudo *R* ^2^	0.399					

*Note:* *, **, and ***Indicates 10%, 5%, and 1% level of significance.

Abbreviation: HH, household head.

### 3.4 Determinants of Crop Diversification: An Ordered Probit Model Estimates

Crop diversification is vital for haor households to increase crop intensification, household income, and climate change adaptation (Musafir et al. 2022; Khan et al. 2017). The results of the Ordered Probit model, which reflect the drivers that influence the extent and magnitude of crop diversification, are shown in Table 6. The overall model fit statistics show that the Ordered Probit model was statistically significant (Wald χ^2^(17) = 118.37, *p* < 0.001), indicating that the explanatory variables together have a statistically significant effect on the ordered crop diversification categories. The model, estimated with 240 observations, had a log pseudolikelihood of −198.81 and a pseudo *R* ^2^ of 0.2384, indicating sufficient explanatory power for cross‐sectional ordered response data. In terms of household characteristics, household head education, dependency ratio, and farm size were positively and significantly associated with crop diversification. The positive coefficient for household head education indicates that more educated farmers were more likely to achieve higher levels of crop diversification. The marginal effects indicated that one additional year of the household head increased the probability of being in fair, good, excellent crop diversification group by 0.8, 0.6, and 0.8 percentage points respectively, while decreased the probability of being in a poor‐diversified category by 2.20 percentage points. This result aligns with prior research showing that enhanced management abilities, education, and training better equip farmers to manage more diverse farms (Bravo‐Ureta et al. 2006; Ashfaq et al. 2008; Islam et al. 2024). The coefficient of the dependency ratio was positive and statistically significant, indicating that the extent of crop diversification was significantly influenced by household dependency burden. However, the marginal effect showed that a one‐unit increase in the dependency ratio increased the probability of achieving fair, good, and excellent crop diversification categories by 1.9, 1.4, and 2.0 percentage points, respectively, while lowering the likelihood of falling into the poor crop diversification category by 5.3 percentage points. The positive marginal effect indicates that households with a larger dependency ratio are more likely to achieve higher crop diversification. It may also indicate farmers' efforts to diversify crop output to increase household food security and stabilize revenue in response to rising household consumption needs. This explanation aligns with Makate et al. (2016) and Gebiso et al. (2023), who claimed that crop diversification is a risk management strategy that improves household income. Farm size showed a positive and highly significant effect on the extent and breadth of crop diversification, indicating that larger landholdings increased the households' likelihood to attain greater crop diversification. The marginal effects revealed that a one‐hectare increase in farm size lowered the likelihood of falling into the poor crop diversification category by 7.0 percentage points, while increased the likelihood of falling into the fair, good, and excellent categories by 2.5, 1.9, and 2.6 percentage points, respectively. Larger farms provide greater flexibility in allocating land among different crops, thereby facilitating crop diversification. This finding in line with the previous studies revealed that farm size positively and significantly affected crop diversification (Benin et al. 2004; Ashfaq et al. 2008; Abay et al. 2009; Bonham et al. 2012; Kanyua et al. 2013; Mussema et al. 2015; Makate et al. 2016; Dessie et al. 2019; Rahman et al. 2026). Among the institutional and infrastructural factors, agricultural training, agricultural marketing training, and extension contact were positively and significantly associated with crop diversification, whereas nearby road distance was negatively and significantly associated with crop diversification. Agricultural training had a positive and significant influence on the extent and magnitude of crop diversification, indicating that involvement in agricultural training enhanced the probability of obtaining a higher level of crop diversification (Nahar et al. 2024). The marginal effects showed that participation in agricultural training reduced the probability of falling into the poor crop diversification group by 16.4 percentage points, while increasing the probability of being in the fair, good, and excellent categories by 5.9, 4.3, and 6.2 percentage points, respectively. This finding suggests that agricultural training improves farmers' knowledge and technical skills, allowing them to adopt more efficient production practices and diversify their cropping systems (Dey 2020; Rashid et al. 2024). Likewise, agricultural marketing training had a positive and statistically significant influence on crop diversification, demonstrating that improved market knowledge enables farmers to cultivate a wider range of crops. The marginal effects showed that farmers who received agricultural marketing training were 5.2, 3.9, and 5.5 percentage points more likely to achieve fair, good, and excellent levels of crop diversification, respectively, while they were 14.6 percentage points less likely to belong to the poor crop diversification group. This finding aligns with Kanchan and Singh (2024) and Nahar et al. (2024), who suggested that agricultural marketing training improves farmers' understanding of market demand, price information, and marketing opportunities, thereby encouraging the cultivation of a wider range of crops. Extension contact had a positive and substantial effect on the extent and level of agricultural diversification, implying that frequent extension services encourage farmers to embrace a wider range of crops. Contact with extension personnel increased the probabilities of belonging to the fair, good, and excellent crop diversification categories by 4.5, 3.4, and 4.8 percentage points, respectively, while lowering the probability of belonging to the poor crop diversification category by 12.6 percentage points. This finding is in line with those of Emmanuel et al. (2016), Anang et al. (2020), and Musafir et al. (2022), who also found a favorable relationship between extension services and crop diversity. They showed that regular contact with extension staff increases farmers' access to technical knowledge, production information, and improved farming practices, therefore boosting the adoption of diverse cropping systems. The coefficient of nearby road distance was negative and statistically significant, indicating that low road accessibility limited farmers' capacity to diversify their farming systems. The marginal effects showed that a one‐kilometer increase in the distance from a household to the nearest road increased the probability of belonging to the poor crop diversification category by 14.3 percentage points, while decreasing the probabilities of belonging to the fair, good, and excellent crop diversification categories by 5.1, 3.8, and 5.4 percentage points, respectively. Proximity to roads facilitates crop diversification by improving farmers' access to input and output markets, reducing transportation costs, and expanding marketing opportunities. In addition to lowering marketing expenses and facilitating speedy produce disposal, a road makes marketplaces more accessible (Shamdasani 2021). Moreover, it reduces the possibility of post‐harvest losses for perishable items (Joshi et al. 2003; Khatun and Roy 2014; Rahman et al. 2026). Among the geographical and climatic factors, the duration of waterlogging and location in Mithamoin upazila were negatively and significantly associated with crop diversification. The duration of waterlogging had a negative and significant effect on the extent and magnitude of crop diversification, indicating that prolonged waterlogging impeded households from achieving higher levels of crop diversification. An increase in the average number of months of waterlogging increased the probability of belonging to the poor crop diversification category by 8.3 percentage points, while decreasing the probabilities of falling into the fair, good, and excellent crop diversification categories by 3.0, 2.2, and 3.1 percentage points, respectively. This insight implies that consistent waterlogging shortens the effective growing season, limits the cultivation of non‐rice crops, and reinforces the dominance of Boro rice cultivation in the haor ecosystem (Hoq et al. 2022; Akter and Mazumder 2025; Rahman et al. 2026). Households located in the Mithamoin upazila had a significant positive influence on crop diversification with reference to Nikli upazila. The marginal effects revealed that households in Mithamoin were 14.1 percentage points more likely to fall into the poor crop diversification category, while the probabilities of falling into the fair, good, and excellent crop diversification categories were 5.2, 3.8, and 5.1 percentage points, respectively. This could be due to the more extensive and prolonged waterlogging in Mithamoin, which limits the cultivation of non‐rice crops and reduces opportunities for crop diversification (Dube and Guveya 2016). Because, low land areas are more prone to waterlogging due to flood and flash flood in the haor areas. This finding is consistent with Hoq et al. (2022) and Islam et al. (2023).

**TABLE 6:** Factors influencing the degree of crop diversification.

Extent and magnitude of crop diversification	Coefficient	*p*	Marginal effect							
			Poor		Fair		Good		Excellent	
			*dy*/*dx*	*p*	*dy*/*dx*	*p*	*dy*/*dx*	*p*	*dy*/*dx*	*p*
Household characteristics										
Age of the HH	0.008	0.262	−0.002	0.263	0.001	0.275	0.001	0.256	0.001	0.273
Education of the HH	0.083***	0.001	−0.022***	0.000	0.008***	0.001	0.006***	0.003	0.008***	0.001
Educated family member	−0.091	0.153	0.024	0.149	−0.009	0.170	−0.006	0.155	−0.009	0.148
Dependency ratio	0.197*	0.051	−0.053**	0.050	0.019*	0.063	0.014*	0.059	0.020*	0.057
Farm size	0.259***	0.000	−0.070***	0.000	0.025***	0.001	0.019***	0.001	0.026***	0.000
Infrastructural and institutional factors										
Agricultural training	0.607***	0.005	−0.164***	0.004	0.059***	0.005	0.043***	0.010	0.062***	0.010
Agricultural marketing training	0.542**	0.016	−0.146**	0.013	0.052**	0.023	0.039**	0.029	0.055***	0.011
Organizational membership	0.035	0.881	−0.010	0.881	0.003	0.881	0.003	0.880	0.004	0.881
Extension contact	0.469**	0.023	−0.126**	0.018	0.045**	0.017	0.034**	0.025	0.048**	0.036
Access to credit	0.083	0.661	−0.023	0.662	0.008	0.662	0.006	0.663	0.008	0.663
Nearby road distance	−0.532**	0.021	0.143**	0.016	−0.051**	0.013	−0.038**	0.032	−0.054**	0.029
Own irrigation facilities	0.023	0.902	−0.006	0.902	0.002	0.902	0.002	0.902	0.002	0.902
Own transport facilities	0.367	0.117	−0.099	0.109	0.035	0.123	0.026	0.115	0.037	0.117
Geographical and climatic factors										
Flood management training	−0.292	0.208	0.078	0.207	−0.028	0.213	−0.021	0.214	−0.030	0.217
Duration of waterlogged	−0.309***	0.007	0.083***	0.006	−0.030***	0.015	−0.022**	0.008	−0.031***	0.008
Agro‐ecological settings	Nikli Upazila is a base category									
Mithamoin	−0.530**	0.030	0.141**	0.025	−0.052**	0.030	−0.038**	0.036	−0.051**	0.038
Itna	−0.159	0.431	0.044	0.430	−0.014	0.421	−0.012	0.433	−0.018	0.443
/cut1	−0.269									
/cut2	0.798									
/cut3	1.488									
Model fit statistics										
Number of observation	240									
Log pseudolikelihood	−198.81									
Wald *χ* ^2^(17)	118.37									
Prob > *χ* ^2^	0.000									
Pseudo *R* ^2^	0.2384									

*Note:* *, **, and ***Indicates 10%, 5%, and 1% level of significance.

Abbreviation: HH, household head.

## 4 Conclusions and Policy Recommendations

This study explored the patterns, extent, and determinants of crop diversification among farm households in the haor areas of Kishoreganj district, Bangladesh. Crop diversification was measured using the Simpson Diversification Index (SDI), while the Censored Tobit and Ordered Probit models were employed to identify the factors associated with crop diversification. Although crop diversification in haor areas remains low, farmers have begun to cultivate mustard, green chili, maize, jute, potato, pumpkin, black gram, cucumber, and groundnut in addition to the dominant Boro rice crop. This gradual diversification has the potential to boost farm income, create employment opportunities, and improve farmers' livelihoods. This study investigated the household, institutional, infrastructural, and climatic factors associated with crop diversification among smallholder farmers in the haor areas of Bangladesh. The findings suggest that improving the significant determinants identified in this study is essential for promoting crop diversification in the haor ecosystem. As a result, the following policy recommendations are proposed to strengthen both the sustainability and resilience of agricultural systems in the haor ecosystem. Household head education, extension contact, and capacity building training facilities should be strengthened, as higher education, regular extension contact, agricultural, and marketing training significantly enhance farmers' technical knowledge of crop production and market orientation. Farm size was positively associated with crop diversification, indicating the need for land availability and improved land‐use management practices for smallholders. Conversely, factors that have negative associations with crop diversification, such as greater distances to nearby roadways, prolonged seasonal waterlogging, and agro‐ecological setting, underscore the importance of improving rural infrastructure, strengthening water management systems, and implementing location‐specific agricultural policies. Improving rural road infrastructure and market connectivity would facilitate access to inputs and markets, thereby encouraging diversification into high‐value crops. In addition, investments in water management, drainage facilities, and the dissemination of short‐duration and flood‐tolerant crop varieties are essential to reduce the adverse effects of prolonged seasonal waterlogging. Research institutions should develop and promote short‐duration, flood‐tolerant crop varieties that are suitable for the waterlogged and agro‐ecological conditions of the Haor ecosystem. Household‐level demonstration programs should also be extended to inspire farmers to adopt these crops within the existing cropping system. These interventions would increase crop diversification, strengthen farmers' resilience to climate‐related risks, and contribute to long‐term agricultural development and food and nutrition security in the Haor region. The current findings should be interpreted with caution due to purposive site selection, cross‐sectional data, self‐reported environmental exposure, and sparse observations in some categories. Nonetheless, the study provides important context‐specific evidence for policymakers, and future research should use longitudinal data, larger probability‐based samples, and alternative econometric methods to address potential endogeneity for sustainable crop diversification in flood‐prone wetland ecosystems.

## Funding

The study was funded by the Bangladesh Agricultural Research Institute (BARI), but the funder had no role in the decision to submit the work for publication.

## Conflicts of Interest

The authors declare no conflicts of interest.

## Supporting information


**File S1:** Dataset used for the analysis of crop diversification.

**File S2:** Stata commands used for data management and statistical analysis.

## Data Availability

The data and Stata analysis scripts supporting the findings of this study are openly available in Zenodo (Hoq et al. 2026, Version 3; https://doi.org/10.5281/zenodo.23375916). The supporting dataset and analysis scripts are also provided as Files S1 and S2, respectively.

## Appendix A

**TABLE A1:** Variance Inflation Factors (VIF) of the independent variables used in the model.

Variables name	VIF	1/VIF
Age of the HH	1.162	0.86
Education of the HH	1.462	0.684
Educated family member	1.388	0.72
Dependency ratio	1.123	0.89
Farm size	1.379	0.725
Agricultural training	1.539	0.65
Agricultural marketing training	1.256	0.796
Organizational membership	1.304	0.767
Extension contact	1.222	0.818
Access to credit	1.21	0.827
Nearby road distance	1.196	0.836
Own irrigation facilities	1.186	0.843
Own transport facilities	1.065	0.939
Flood management training	1.341	0.746
Duration of waterlogging	1.23	0.813
2. Upazilla code (Mithamoin)	1.6	0.625
3. Upazilla code (Itna)	1.681	0.595
Mean VIF	1.314	.

**TABLE A2:** Correlation matrix of the independent variables.

Variables	(1)	(2)	(3)	(4)	(5)	(6)	(7)	(8)	(9)	(10)	(11)	(12)	(13)	(14)	(15)	(16)
(1) Age of the HH	1															
(2) Education of the HH	−0.211	1														
(3) Educated family member	0.086	0.34	1													
(4) Dependency ratio	−0.127	0.05	−0.097	1												
(5) Farm size	−0.006	0.279	0.332	0.033	1											
(6) Agricultural training	−0.074	0.258	0.278	−0.015	0.353	1										
(7) Agricultural marketing training	−0.059	0.174	0.08	0.078	0.083	0.248	1									
(8) Organizational membership	0.024	0.159	0.063	−0.014	0.082	0.284	0.388	1								
(9) Extension contact	−0.063	0.155	0.166	−0.02	0.143	0.32	0.091	0.117	1							
(10) Access to credit	−0.077	0.053	0.15	−0.019	0.153	0.182	0.078	0.042	0.185	1						
(11) Nearby road distance	−0.011	−0.121	−0.066	0.031	−0.179	−0.226	−0.087	−0.092	−0.036	−0.137	1					
(12) Own irrigation facilities	−0.016	0.062	0.019	0.042	0.222	0.131	0.077	0.051	0.131	0.038	0.124	1				
(13) Own transport facilities	−0.043	0.063	0.175	−0.062	0.059	0.04	0.022	0.093	−0.025	−0.017	−0.038	−0.045	1			
(14) Flood management training	−0.032	0.347	0.085	0.034	0.096	0.279	0.144	0.194	0.066	−0.019	−0.056	−0.042	0.074	1		
(15) Duration of waterlogging	0.062	−0.12	−0.135	−0.033	−0.081	−0.163	−0.176	−0.159	−0.244	−0.192	0.089	−0.082	−0.057	−0.274	1	
(16) Agro‐ecological settings	0.112	0.011	−0.018	0.174	0.096	−0.048	0.108	0.139	−0.036	−0.255	0.149	0.061	0.034	−0.028	0.004	1

**TABLE A3:** Tests for heteroskedasticity in the Tobit model.

Diagnostic test	*χ* ^2^	df	*p*	Decision
Breusch‐Pagan/Cook‐Weisberg test	6.93	1	0.008	Reject H_0_: Constant variance (homoscedasticity)
White's test	170.9	159	0.245	Do not reject H_0_: Constant variance (homoscedasticity)
