# Supplementary material for: Determinants of Crop Diversification in Boro Rice‐Based, Wetland Farming Systems in Bangladesh
Source: Plant Environ Interact. 2026 Oct 28;7(6):e70227. doi: 10.1002/pei3.70227 (PMC13716940; doi:10.1002/pei3.70227)
Supplement: Supplementary file 1 [file PEI3-7-e70227-s002.docx]

Stata script for data analysis

summarize HH_age HH_edu Educated_FM Dependancyratio Farm_size_ha Ag_training Ag_mrkting_training Organizational_membership Extension_linkage Ag_credit_facilities Home_road_distnce Own_irrigation_facilities Own_transport_facilities flood_managemant_training Months_waterlogging Upazilla_code

tobit SDI HH_age HH_edu Educated_FM Dependancyratio Farm_size_ha Ag_training Ag_mrkting_training Organizational_membership Extension_linkage Ag_credit_facilities Home_road_distnce Own_irrigation_facilities Own_transport_facilities flood_managemant_training Months_waterlogging ib(1).Upazilla_code,ll(0) vce(robust)

margins, dydx(*) predict(ystar(0,.))

oprobit rder_SDI HH_age HH_edu Educated_FM Dependancyratio Farm_size_ha Ag_training Ag_mrkting_training Organizational_membership Extension_linkage Ag_credit_facilities Home_road_distnce Own_irrigation_facilities Own_transport_facilities flood_managemant_training Months_waterlogging ib(1).Upazilla_code, vce(robust)

margins, dydx(*) predict(outcome(1))

margins, dydx(*) predict(outcome(2))

margins, dydx(*) predict(outcome(3))

margins, dydx(*) predict(outcome(4))

reg SDI HH_age HH_edu Educated_FM Dependancyratio Farm_size_ha Ag_training Ag_mrkting_training Organizational_membership Extension_linkage Ag_credit_facilities Home_road_distnce Own_irrigation_facilities Own_transport_facilities flood_managemant_training Months_waterlogging ib(1).Upazilla_code

vif

estat hettest

estat imtest, white

corr HH_age HH_edu Educated_FM Dependancyratio Farm_size_ha Ag_training Ag_mrkting_training Organizational_membership Extension_linkage Ag_credit_facilities Home_road_distnce Own_irrigation_facilities Own_transport_facilities flood_managemant_training Months_waterlogging Upazilla_code

histogram SDI, width(0.04) start(0) density xlabel(0(.1)1, labsize(medium) format(%3.1f)) xtitle("Simpson's Diversification Index (SDI)", size(medium)) ytitle("Density", size(medium)) title("A. Distribution of SDI", size(large))xline(0.25 0.50 0.75, lpattern(dash) lwidth(medium))saving(hist_sdi.gph, replace)

kdensity SDI, xlabel(0(.1)1, labsize(medium) format(%3.1f)) xtitle("Simpson's Diversification Index (SDI)", size(medium)) ytitle("Density", size(medium))title("B. Kernel Density Estimate", size(large)) xline(0.25 0.50 0.75, lpattern(dash) lwidth(medium))saving(kdensity_sdi.gph, replace)

graph combine hist_sdi.gph

kdensity_sdi.gph,cols(1)imargin(small)title("Distribution of Original SDI (0–1)", size(large))graphregion(color(white))xsize(7) ysize(10)

graph export "SDI_distribution_highres.tif", replace width(4000)
